# Regulation of the master regulator FOXM1 in cancer

**DOI:** 10.1186/s12964-018-0266-6

**Published:** 2018-09-12

**Authors:** Guo-Bin Liao, Xin-Zhe Li, Shuo Zeng, Cheng Liu, Shi-Ming Yang, Li Yang, Chang-Jiang Hu, Jian-Ying Bai

**Affiliations:** Department of Gastroenterology, Xinqiao Hospital, Third Military Medical University (Army Medical University), Chongqing, 400037 China

**Keywords:** FOXM1, Regulation, Transcriptional, Post-transcriptional, Post-translational

## Abstract

FOXM1 (forkhead box protein M1) is a critical proliferation-associated transcription factor that is widely spatiotemporally expressed during the cell cycle. It is closely involved with the processes of cell proliferation, self-renewal, and tumorigenesis. In most human cancers, FOXM1 is overexpressed, and this indicates a poor prognosis for cancer patients. FOXM1 maintains cancer hallmarks by regulating the expression of target genes at the transcriptional level. Due to its potential role as molecular target in cancer therapy, FOXM1 was named the Molecule of the Year in 2010. However, the mechanism of FOXM1 dysregulation remains indistinct. A comprehensive understanding of FOXM1 regulation will provide novel insight for cancer and other diseases in which FOXM1 plays a major role. Here, we summarize the transcriptional regulation, post-transcriptional regulation and post-translational modifications of FOXM1, which will provide extremely important implications for novel strategies targeting FOXM1.

## Background

**Fig. 1:**
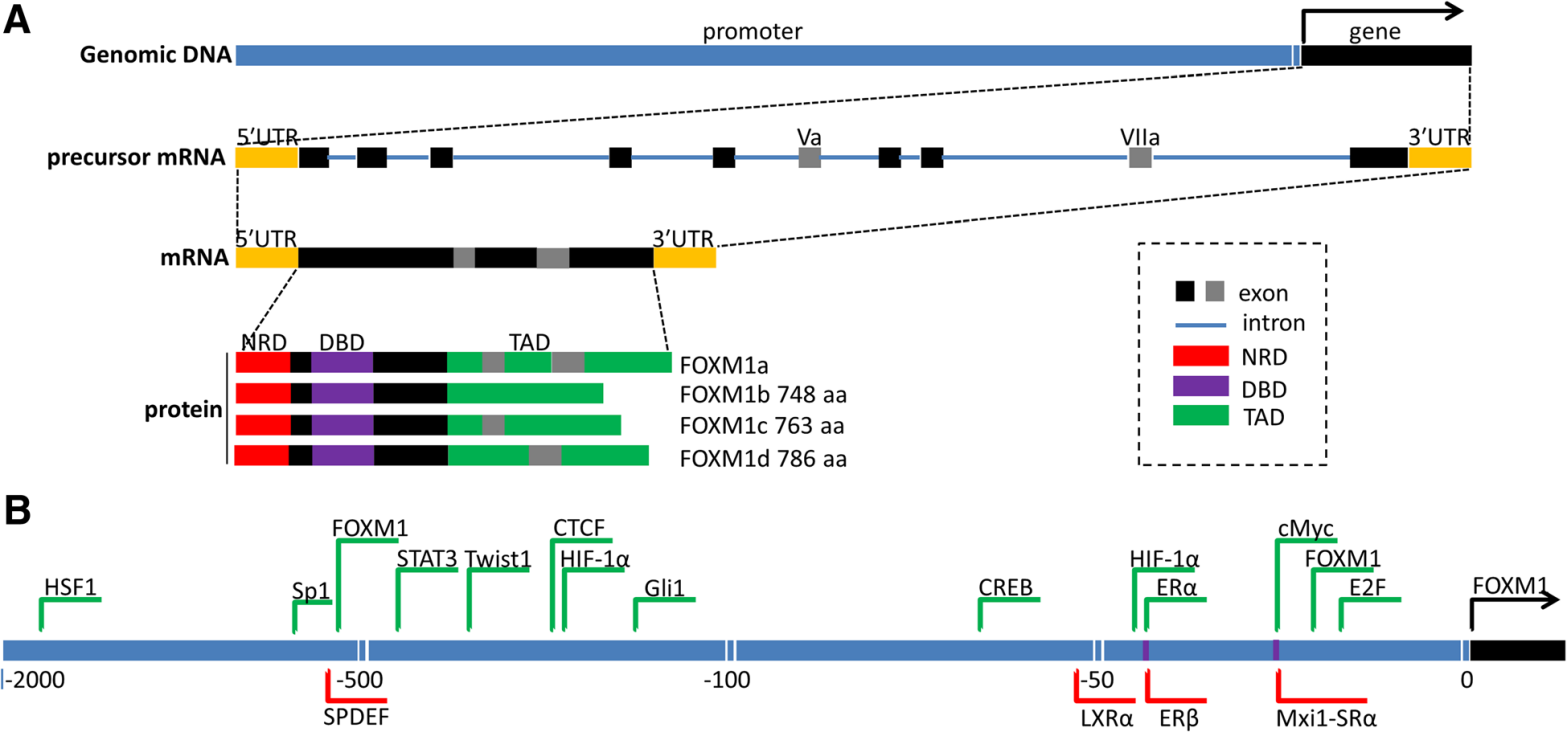
Genomic structure and coding isoforms in the FOXM1 gene and transcription factor binding sites in FOXM1 promoter region. **a**. Genomic structure and coding isoforms of the FOXM1 gene. **b**. Schematic diagram of transcription factor binding sites during FOXM1 promoter region. Green: activator, red: repressor, purple: cis-acting element could be bound by both activator and repressor

Forkhead box M1 (FOXM1), previously named HNF-3, HFH-11 or Trident, is a transcription factor of the Forkhead box (Fox) protein superfamily which is defined by a conserved winged helix DNA-binding domain1 [1]. The human FOXM1 gene consists of 10 exons, of which exons Va and VIIa can be alternatively spliced [2]. In the past, VIIa was treated as a repressor until a novel isoform (FOXM1d) that could promote the epithelial–mesenchymal transition (EMT) and metastasis by activating ROCKs in colorectal cancer was identified [3, 4]. Accordingly, there are four isoforms of human FOXM1 identified to date (Fig. 1a). FOXM1a contains both exons Va and VIIa and lacks transactivation activity, while the rest of the three, FOXM1b (which contains neither exon Va nor VIIa), FOXM1c (no VIIa) and FOXM1d (no Va) are transcriptionally active. The FOXM1 protein contains a conserved forkhead DNA-binding domain (DBD), an N-terminal repressor domain (NRD), and a C-terminal transactivation domain (TAD). The transactivation activity of TAD can be suppressed by direct interaction with the NRD [5, 6]. In addition, murine FOXM1 splice variants display the same DNA-binding specificity as human FOXM1 and bind to DNA-binding sites with the consensus sequence 5′-A-C/T-AAA-C/ T-AA-3’ [7]. The study of murine FOXM1 may also be applied to human FOXM1. For example, it has been demonstrated that Gli1 regulates FOXM1 in murine stem cells [8]. In human basal cell carcinomas and colorectal cancer cells, FOXM1 is also a direct target of Gli1 [9, 10]. This may be due to the evolutionary conservation between the DNA binding domain of both human and murine FOXM1, suggesting the FOXM1 of the two species may share target genes. As such, investigating the regulation of murine FOXM1 may provide significant implications into the dysregulation of human FOXM1. Furthermore, a mouse model is a suitable experimental model for the development of novel FOXM1 inhibitors.

**Fig. 2:**
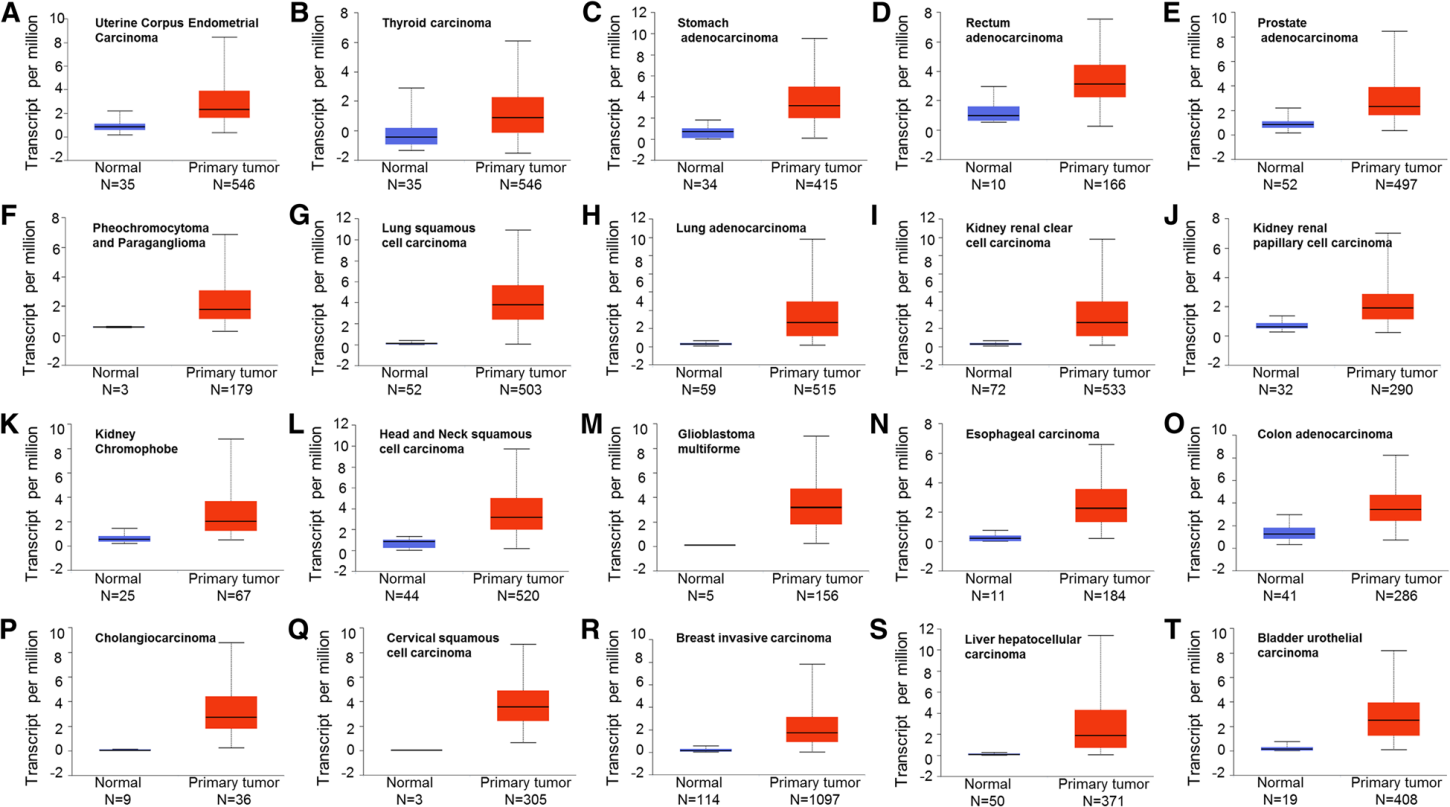
FOXM1 expression profile from the TCGA database. The FOXM1 transcript per million are presented in differernt cancers and corresponding normal tissues, including ulterine corpus endometrial carcinoma (**a**), thyroid carcinoma (**b**), stomach adenocarcinoma (**c**), rectum adenocarcinoma (**d**), prostate adenocarcinoma (**e**), pheochromocytoma and paraganglioma (**f**), lung squamous cell carcinoma (**g**), lung adenocarcinoma (**h**), kidney renal clear cell carcinoma (**i**),kidney renal papillary cell carcinoma (**j**), kidney chromophobe (**k**),  head and neck squamous cell carcinoma (**l**), glioblastoma multiforme (**m**), esophageal carcinoma (**n**), colon carcinoma (**o**), cholangiocarcinoma (**p**), cervical squamous carcinoma (**q**), breast invasive carcinoma (**r**), liver hepatocellular carcinoma (**s**), bladder urothelial carcinoma (**t**)

**Fig. 3:**
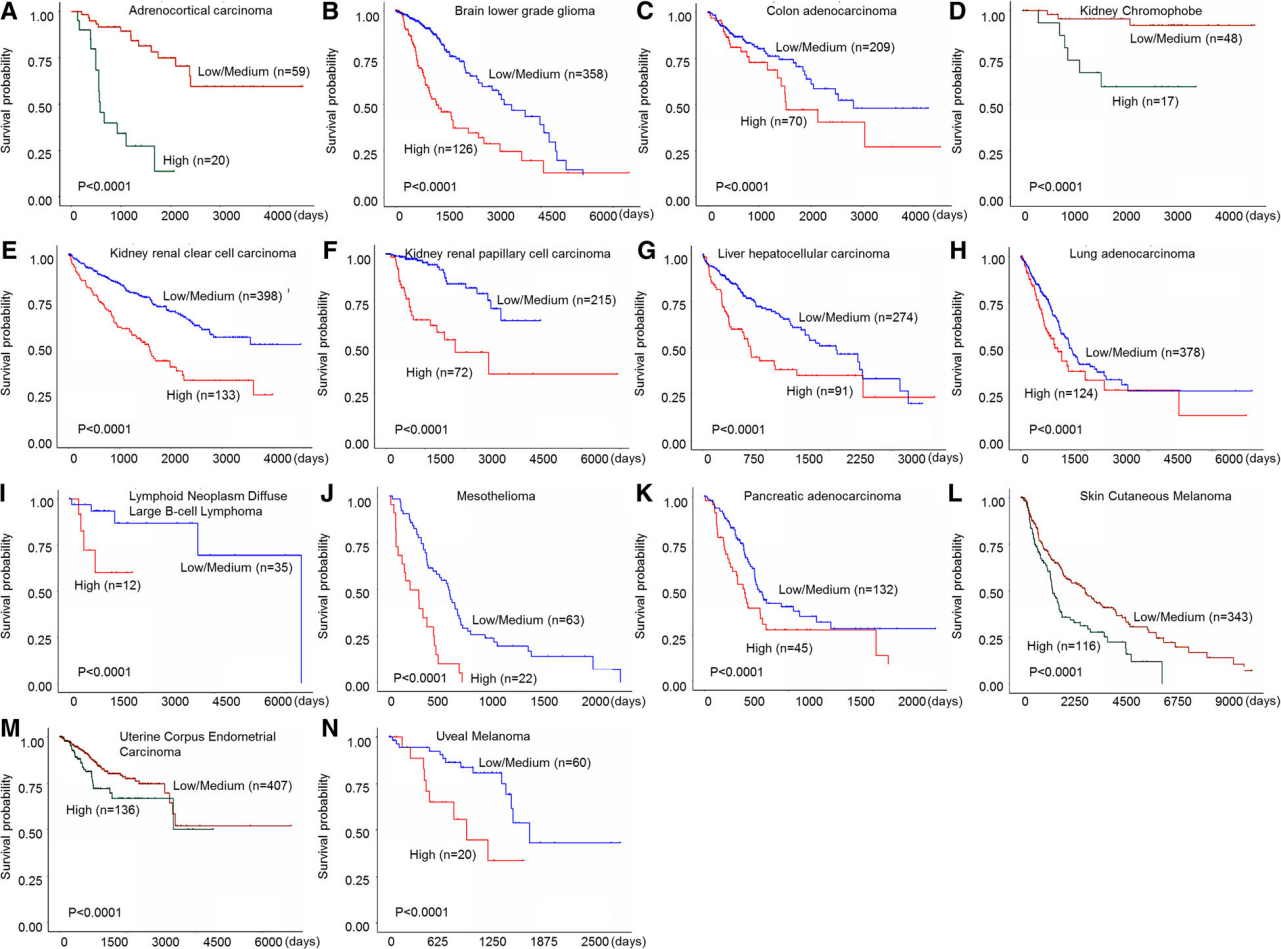
Kaplan–Meier analyses of overall survival according to FOXM1 expression levels in different cancer suffers, including adrenocortical carcinoma (**a**), glioma (**b**), colon adencarcinoma (**c**), kidney chromophobe (**d**),kidney renal clear cell carcinoma (**e**),kidney renal papillary cell carcinoma (**f**),liver hepatocellular carcinoma (**g**),lung adenocarcinoma (**h**),ulterine corpus endometrial carcinoma (**m**),uveal melanoma (**n**)

FOXM1 is detected primarily in progenitor and regenerating tissues, as well as tumor cells, which are all highly proliferative [11]. As a classic proliferation-associated transcription factor, FOXM1 directly or indirectly activates the expression of target genes at the transcriptional level and exhibits a spatiotemporal pattern whose dysregulation is involved in almost all hallmarks of tumor cells [3, 12]. Increased expression of FOXM1 is observed in a variety of human cancers, such as ovarian cancer, breast cancer, prostate cancer, hepatoma, angiosarcoma, colorectal cancer, melanoma, lung cancer, and gastric cancer [13–21], which is consistent with the results obtained from the TCGA database (Fig. 2). Inhibition of FOXM1 in cancer cells leads to decreased cell proliferation and migration, metastasis, angiogenesis, EMT, and drug resistance [22–26]. Furthermore, a recent meta-analysis revealed that elevated FOXM1 expression is related to poor prognosis in most solid tumors [27], which is also further confirmed by the TCGA database (Fig. 3) [28]. These results clearly showed the important role of FOXM1 in tumorigenesis and cancer development. Therefore, FOXM1 has been identified as a potential therapeutic target for the treatment of cancers. Although a few drugs and inhibitors have been shown to be effective at inhibiting the activity of FOXM1 in vitro, they’ve yet to pass successfully into clinical use [29]. This is likely due in major part to the poor current understanding of the regulation of FOXM1. Hence, a comprehensive review of FOXM1 regulation will thus contribute to the extensive effort and research into the gene as a therapeutic target for a number of FOXM1-dependent conditions, such as the cancers mentioned previously.

In this review, we provide an overview of how FOXM1 is regulated and focus on the transcriptional, post-transcriptional, post-translational, and protein-protein/RNA interaction levels. Though many biomolecules regulate the expression of FOXM1, we emphasize the biomolecules that directly interact with or modify the promoter, RNA, or protein of FOXM1. At the same time, we will discuss briefly the pharmacological inhibition of FOXM1.

## Transcriptional regulation of FOXM1

The core promoter regions of the FOXM1 gene contain several classic regulatory elements, such as E-boxes, and other cis-acting elements that can function as responsive elements to other transcription factors. Here, we mainly discuss the transcription factors that directly bind to the promoter regions (which are verified by electrophoretic mobility shift assays and/or chromatin immunoprecipitation assays) (Fig. 1b).

Most responsive elements are located adjacent to FOXM1’s transcriptional start sites, though the furthest element is found approximately 2000 bp away. More than 75% of these binding sites act as cis-activation elements, but they do not all function through the same basic mechanisms. For example, glioma-associated oncogene homolog 1 (Gli1), CCCTC-binding factor (CTCF), cAMP responsive element-binding protein (CREB), signal transducer and activator of transcription 3 (STAT3), and E2F interact directly with their binding sites and upregulate FOXM1 expression [9, 30–34]. In human colorectal cancer (CRC), Gli1 regulates FOXM1 by directly binding to its promoter at BS4 (GCCCACCCA), which contributes to the proliferation of CRC cells [9]. The DNA-binding protein, CTCF, may regulate the motility and invasiveness of primary hepatocellular carcinoma (HCC) cells via interaction with the CTCF-binding site(s) located in the proximal promoter of FOXM1 [30]. In HBV-associated HCC, CREB directly bound to the FOXM1 promoter in response to HBx and facilitates hepatoma cell invasion and metastasis [33]. In the chronic myeloid leukemia K562 cell line, FOXM1 is transcriptionally dependent on STAT3 and crucial for its cell proliferation, cell cycle checkpoints and viability [32]. However, twist-related protein 1 (Twist1) may not be sufficient to enhance FOXM1 gene transcription unless it recruits the coactivator p300 to form a complex. In gastric cancer, Twist 1 could bind to the promoter region of FOXM1, and subsequently recruit p300 to induce its mRNA transcription [35]. Other transcription factors are stress-dependent. For example, under normoxic conditions, HIF-1α is unstable because specific proline residues are hydroxylated. Under hypoxic conditions, HIF-1α directly binds to the promoter of FOXM1 and activates its expression. Consequently, the upregulation of FOXM1 promotes the proliferation of hepatoma cells and enhances their resistance to apoptosis [36, 37]. Moreover, Dai B et al. reported that upon heat shock stress HSF1 was released from Hsp90 and translocated from cytoplasm to nucleus. Then HSF1 directly bound to FOXM1 promoter and increased FOXM1 promoter activity [38]. They also revealed that overexpression of a constitutively active HSF1 up-regulated FOXM1 expression in Hs683 cells, indicating that HSF1 may also regulate FOXM1 expression in glioma cell lines under normal conditions [38]. Taken together, the evidence suggests that HSF1 regulates the FOXM1 expression under both heat shock stress and normal conditions.

The FOXM1 promoter region also contains sites that can function as cis-suppression elements. For example, liver X receptor a (LXRa) functions as a transcriptional repressor for FOXM1 expression by binding to an inverted repeat IR2 (-52CCGTCAcgTGACCT-39) in the FOXM1 promoter region, and suppresses the proliferation of HCC cells [39]. Barsotti AM et al. reported that p53-mediated inhibition of FOXM1 is partially p21 and retinoblastoma (RB) family dependent in MCF-7 cells [40], while FOXM1 was demonstrated as direct target gene of p53 in mice hepatocytes [41]. Some responsive elements have dual roles, such as the ERE (estrogen-response element) and the E-box within the proximal promoter region of the FOXM1 gene. In ERα-positive breast cancer cells, ERα can upregulate FOXM1 expression by binding to the ERE. Conversely, ERβ binding to the ERE down-regulates FOXM1 expression [42, 43]. In a similar fashion, the E-box is a competitive binding site for c-Myc (activator) and Mxi1-SRα (repressor) [44, 45].

It is worth noting that, there exists a positive auto-regulation loop of FOXM1. In 2009, Marianna Halasi first found that siomycin A and thiostrepton, transcriptional inhibitors of FOXM1, downregulated both the transcriptional activity and expression levels of FOXM1 [46]. The phenomenon indicates the existence of an auto-regulation loop for FOXM1. Subsequently, a research study showed that disruption of FOXM1 binding site inhibited FOXM1 promoter activity, confirming that the − 745/− 738 bp region is required for the auto-regulatory activation of the FOXM1 promoter [47, 48]. Furthermore, it has been reported that nuclear AURKA can be recruited by FOXM1 as a co-factor to transactivate FOXM1 target genes in a kinase-independent manner [49]. AURKA and FOXM1 participated in a tightly coupled positive feedback loop to enhance BCSC phenotype. Moreover, AURKA can effectively transactivate the FOXM1 promoter through a Forkhead response element, whereas FOXM1 can activate AURKA expression at the transcriptional level [49]. All these findings confirm the existence of an autoregulatory loop, suggesting that FOXM1 protein can potentially bind to FOXM1 promoter region.

Herein, we have summarized all the reported transcription activators and repressors that directly bound to FOXM1 core promoter (Table 1). This will not only benefit for the discovery of novel transacting factors during the regulatory region of FOXM1 promoter, but it will also provide important implications for the design of drugs targeting FOXM1.

**Table 1:** Factors reported to bind directly to FOXM1 promotor and regulated its expression

Transcription factor	Responsive element	Act. / Rep.	Cell	Coactivator	Pos. / Neg. regulation
cMyc [109, 110]	E-Box	A	U-937 etc.		CAR [44]/−
Mxi1-SRα [45]	E-Box	R	DL23		-/FOXO3a [111]
HIF-1α [36]	−271/− 267, − 47/− 42	A	HepG2		TNF-α [37]/−
ERα [42]	−45	A	MCF-7 ZR-75-1		-/HDACs
ERβ [43]	−45	R	MCF-7		
E2F [34]	−24	A	MCF-7		MnSOD [31]/P53 [34, 112]
STAT3 [32]	−440/− 432	A	K562		MAPK/−
CREB [33]	−60/− 36	A	HepG2		HBx/−
HSF1 [38]	− 1792/− 1767	A	U-87MG		
Twist1 [35]	− 375/− 352	A	NCI-N87	P300	
LXRa [39]	−52/− 39	R	HepG2		
Gli1 [9, 10]	−216/− 204	A	HT29		
Gli1, Gli2 [8]	Not clear	A	Murine NSCs		
SPDEF [47]	−670/− 660	R	TRAMP-C2R3		
FOXM1 [46, 47]	−745/− 660, − 27/− 22	A	TRAMP-C2R3, MCF-7		AURKA [49]/−
CTCF [30]	− 296/− 120	A	HepG2		
Sp1 [95]	− 891	A	HepG2		
P53 [41]	Not clear	R	mice hepatocyte		

*Act. /Rep*. Activator or repressor, *Pos. /Neg.* positive or negative regulatory factor, *A* activate, *R* repress

## Post-transcriptional regulation of FOXM1

In addition to the normal processes of post-transcriptional splicing and modification, there are other mechanisms by which FOXM1 can be regulated post-transcriptionally. For example, there are a number of non-coding RNAs (ncRNAs) considered to be important in this regulation. MicroRNAs (miRNAs) are endogenous, highly conserved, non-coding RNAs of approximately 21–24 nucleotides that can guide mRNA degradation or repress translation by binding to complementary sequences in the 3′ untranslated regions (3’UTRs) of targeted mRNAs [50]. Long non-coding RNAs (lncRNAs) are ncRNAs longer than 200 nucleotides that can act as competing endogenous RNAs (ceRNAs). The ceRNAs, known as miRNA “decoys” or “sponges”, are RNA transcripts that competitively bind to the same miRNA via base pair complementarity with miRNA recognition/response elements (MREs) [51]. MicroRNAs and lncRNAs regulate each other through the binding sites of their response elements (MREs) [52, 53].

To date, dozens of miRNAs have been found to regulate the proliferation, invasion, migration, senescence, apoptosis, epithelial-mesenchymal transition (EMT), and drug sensitivity of cancer cells through binding to the 3’UTRs of FOXM1 mRNA. For example, miRNA-214 acts as a tumor repressor during the process of migration, and invasion, and is associated with sensitivity to cisplatin in cervical cancer via directly binding to the 3’UTRs of FOXM1 mRNA [54]. MirRNA-149 can inhibit EMT in non-small cell lung cancer cells (NSCLC cells) by the same mechanism (Table 2) [55]. Compared with miRNAs, few lncRNAs have been identified to regulate FOXM1 expression. In gallbladder cancer (GBC), LncRNA H19 upregulates FOXM1 expression and promotes its proliferation and invasion, through competitively ‘sponging’ miR-342-3p [56, 57]. Another lncRNA, colon cancer-associated transcript 2 (CCAT2), upregulates FOXM1 expression and promotes HCC cell growth through interaction with and suppression of miR-34a [58].

**Table 2:** Non-coding RNAs interaction with FOXM1 transcript

MicroRNA	mechanism	CeRNA	Physiological context	Cancer
miR-216b	3′UTR		proliferation^b^, invasion^b^	Glioblastoma [113], melanoma [114], hepatocellular carcinoma [115]
MiR-214	3′UTR		proliferation^b^, invasion^b^, drug sensitivity^a^	cervical cancer [54]
miR-361-5p	3′UTR		proliferation^b^, invasion^b^	lung cancer [116]
miR-342	3′UTR		proliferation^b^, migration^b^	colorectal cancer [117]
miR-671-5p	3′UTR		proliferation^b^,invasion^b^,EMT^b^	breast cancer [118]
miR-149	3′UTR		drug sensitivity^a^, EMT^b^	Colorectal Cancer [55], non-small cell lung cancer [119]
miR-509-5p	3′UTR		proliferation^b^, migration^b^, invasion^b^	non-small cell lung cancer [120]
miR-802	3′UTR		proliferation^b^	breast cancer [121]
miR-34a	3′UTR	CCAT2 [58]	senescence^a^	hepatocellular carcinoma [122]
miR-370	3′UTR		proliferation^b^,apoptosis^a^	osteosarcoma [123], laryngeal squamous cell carcinoma [124], gastric carcinoma [125]
miR-877	3′UTR		proliferation^b^	hepatocellular carcinoma [126]
miR-320	3′UTR		drug sensitivity^a^, radiosensitivity^a^	colon cancer [127], Glioma [128]
miR-204	3′UTR		invasion^b^, EMT^b^	esophageal cancer [129]
miR-24-1	3′UTR		proliferation^b^	bladder cancer [130]
miR-342-3p	3′UTR	H19 [57]	proliferation^b^, migration^b^, invasion^b^	cervical cancer [56]

^a^: promoting

^b^: Inhibition

In addition, lncRNAs also regulate nascent FOXM1 transcripts. For example, lncRNA FOXM1-AS interacts with nascent FOXM1 transcripts, promoting the ALKBH5-mediated demethylation and subsequent FOXM1 up-regulation. Besides, with the emerging research of the non-coding RNAs, including the circular RNA, piwi-interacting RNA, snRNA and snoRNA, the possible regulation between these non-coding RNAs and FOXM1 require further study for determination.

## Post-translational modifications of FOXM1

Post-translational modifications (PTMs) are the chemical modifications of a protein after its translation, which can have broad effects on the targets. The FOXM1 protein is modified by multiple PTMs that include phosphorylation, ubiquitination, SUMOylation, acetylation and methylation, which may have activating or inhibiting effects. These modifications determine the cellular localization, protein stabilization, and transcriptional activity of FOXM1 in normal or disease states (Table 3).

**Table 3:** Post-translational modifications of FOXM1

PTM	Enzyme	Effect	Cell cycle	FOXM1 isoform
Phosphorylation				
T620,T627,S672	Cyclin D-CDK4/6[6]	Transcriptional activity^a^ stabilization^a^	G1 → S	FOXM1c
S331	Raf/MEK/MAPK [60]	Nuclear translocation^a^	Late S	FOXM1c
S704	Raf/MEK/MAPK [60]	Transcriptional activity^a^	Late S	FOXM1c
T600,T611,S638	Cyclin A/E-Cdk2 [62, 63]	Transcriptional activity^a^	G2/M	FOXM1c
T596	Cyclin B1-Cdk1 [64]	Transcriptional activity^a^	G2/M	FOXM1b
S678	Cyclin B1-Cdk1 [65]	Transcriptional activity^a^	Late G2	FOXM1b
S507,S657, T585	Cyclin B1-Cdk1 [131]	Transcriptional activity^a^	G2/M	FOXM1b
S715,S724	Plk1 [64]	Transcriptional activity^a^	G2 → M/M	FOXM1b
S361	Chk2 [66]	stabilization^a^	DDR	FOXM1b
S251	^c^ [59]	CDK1-dependent phosphorylation^a^	G2/M	FOXM1b
S474	GSK3 [67]	degradation^a^		FOXM1b
Ubiquitination				
-	APC/C-Cdh1 [71, 72]	degradation^a^	Late M/Early G1	FOXM1
-	CRL4-VprBP [73]	degradation^a^	G1/S	FOXM1
-	SCF/FBXO31 [74]	degradation^a^	G2 → M	FOXM1
-	SCF/FBXL2 [132]	degradation^a^		FOXM1
-(K48)	FBXW7 [67]	degradation^a^		FOXM1
-(K48)	RNF8,RNF168 [75]	degradation^a^	(DDR)	FOXM1
De-ubiquitination				
-	USP5 [67]	Nuclear translocation^a^		FOXM1
(K48)	OTUB1 [76, 77]	degradation^b^		FOXM1
SUMOylation				
Lys132,144,201,218,356,368,415,440,443,460,478,495(SUMO2)	^c^ [78]	Transcriptional activity^a^ Nuclear translocation^a^	G2/M	FOXM1c
Lys463(SUMO1)	PIASy [79]	Transcriptional activity^a^		FOXM1b
Lys201,218,460,478,495(SUMO1)	Ubc9 [80]	Cytoplasmic translocation^a^ Cdh1-mediated degradation^a^	(CDR)	FOXM1c
-(SUMO1,2,3)	Ubc9,PIAS1 [81]	Cytoplasmic translocation^a^ stabilization^b^		FOXM1b
Lys201,218,341,445,462,480(SUMO1)	^c^ [68]	Cytoplasmic translocation^a^ stabilization^b^	Late M	FOXM1b
Acetylation				
Lys63,422,440,603,614	CBP/p300 [82]	stabilization^a^ DNA-binding ability^a^Transcriptional activity^a^	G2/M	FOXM1c
Methylation				
-	SETD3 [83]	Transcriptional activity^b^		FOXM1

*K48* Lys48-linked poly-ubiquitin chains, *DDR* DNA-damage response, *CDR* cytotoxic drug response

^a^: Promoting

^b^: Inhibition

^c^: not clear

→ Transition

### Phosphorylation

The activity of FOXM1 is of great importance to the cell cycle, and phosphorylation of FOXM1 protein plays a key role in that activity. The transcriptional activity of FOXM1 is upregulated through the cell cycle and is consistent with its phosphorylation [59]. With the progress of the cell cycle, FOXM1 phosphorylation is constantly changing. The FOXM1 protein maintains a relative hypo-phosphorylation status in the G1/S phase, exhibits increased phosphorylation from the S phase to the G2/M transition, reaches hyper-phosphorylation status in the M phase, and is subsequently dephosphorylated in the late M phase. This dynamic and tight phosphorylation change is mediated by various kinases and their positive feedback loops.

The transactivation domain (TAD) of FOXM1 can be suppressed by direct interaction with the NRD (N-terminal repression domain). To a transcription factor such as FOXM1, sufficient protein levels, nuclear localization and exposure of the TAD are indispensable for maximizing transcriptional activity. In the G1/S phase, FOXM1 mRNA reaches its peak while the FOXM1 protein exhibits low transcriptional activity due to cytoplasmic localization and NRD inhibition of the TAD [60]. In the late G1 phase, Cyclin D-CDK4/6 complexes phosphorylate multiple sites of FOXM1, including T620, T627, and S672, which then triggers the G1 to S cell cycle transition [6]. Interestingly, during this process, B55α (a subunit of protein phosphatase 2A) prevents premature activation of FOXM1 through contact with FOXM1 and repression of cyclin A-CDK [6, 61]. In the late S and G2/M phases, phosphorylation of both S331 and S704 of FOXM1 via the Raf/MEK/MAPK pathway stimulates FOXM1 nuclear translocation and thus promotes the transcriptional activity of FOXM1 [60]. During the G2 phase, cyclin A/E-CDK2 complexes phosphorylate FOXM1, including sites T600, S638, and especially T611, which relieves repression of TAD by NRD, and restores the TAD transactivation activity [62, 63]. In addition, phosphorylation at S251 is critical for cyclin-B1-Cdk1-dependent phosphorylation of FOXM1 [59]. The phosphorylation at T596 by cyclin-B1-Cdk1 on the one hand recruits Plk1 directly to phosphorylate FOXM1 at S715 and S724, which promotes the transcriptional activity of FOXM1 [64]. On the other hand, that phosphorylation recruits the transcriptional co-activator p300/CREB binding protein (CBP) to enhance its transcriptional activity [65].

In response to DNA damage, checkpoint kinase 2 (Chk2) phosphorylates FOXM1 at S361, inhibiting its degradation and increasing transcription of XRCC1 and BRCA2 genes, which are required for repair of DNA damage [66].

FOXM1 phosphorylation is also linked to ubiquitination and SUMOylation. For example, GSK3 phosphorylates FOXM1 at the S474 site, which promotes its subsequent ubiquitin-mediated degradation by FBXW7 [67]. Plk1-mediated phosphorylation of FOXM1 antagonizes its SUMOylation and facilitates cell cycle progression [68].

### Ubiquitination and de-ubiquitination

Ubiquitin (Ub) is a 76-amino acid protein with seven lysine residues that can conjugate to substrate proteins and form a poly-ubiquitin chain, conferring a range of functions. For example, the K48- and K11-linked poly-ubiquitin chains lead to proteolysis of the substrate protein, while the K63-linked poly-ubiquitin chain functions in signal transduction [69]. Ubiquitination is an enzymatic PTM in which an ubiquitin protein is attached to a target protein. De-ubiquitination opposes the role of ubiquitination by removing ubiquitin from substrate proteins.

The N-terminus of the FOXM1 protein contains KEN box (K-E-N-X-X-X-N) and destruction box (R-X-X-L-X-X-X-X-N) sequences that are involved in its ubiquitin-mediated degradation. The KEN box was first found to be an anaphase promoting complex (APC) recognition signal [70], which is responsible for the Cdh1-APC-mediated ubiquitination. Both the KEN box and destruction box (D box) of FOXM1 can be recognized by some ubiquitin protein ligases. In the late M and early G1 phases,Cdh1 interacts with FOXM1 and recruits APC/C E3 ubiquitin ligases to degrade it, which inhibits cell cycle progression [71, 72]. The E3 ligase system is delicate in its regulation of FOXM1 during the cell cycle. For example, in the G1/S phases, E3 ubiquitin ligase CRL4 integrates with its receptor VprBP, which promotes FOXM1 degradation to maintain its relatively low level. In G2/M phase, when FOXM1 is indispensable, VprBP separates from CRL4, relinquishing its inhibition of FOXM1 [73]. During the G2/M transition, SCF/FBXO31 E3 ubiquitin ligases act as negative regulators of FOXM1 via ubiquitin-mediated degradation, which can maintain genomic stability [74].

FOXM1 ubiquitination is also linked to SUMOylation. For example, RNF168 can modulate DNA-damage response (DDR) by promoting protein ubiquitination. In breast cancer treated with epirubicin, FOXM1 is modified through SUMOylation, which leads to its ubiquitination and degradation by RNF168 E3 ubiquitin ligase [75].

In contrast, deubiquitinating enzymes (DUBs) can remove the poly-ubiquitin chains on FOXM1 protein. For instance, in epirubicin-resistant breast cancer and in ovarian cancer, OTUB1 catalyzes the cleavage of the K48-specific ubiquitin linkage from FOXM1, which promotes cancer progression via facilitating cell proliferation and drug resistance [76, 77].

### SUMOylation

Small Ubiquitin-like Modifier (SUMO) proteins are small proteins that are covalently attached to other proteins and modify their function. In mammals, there are four SUMO isoforms, SUMO-1, SUMO-2, SUMO-3, and SUMO-4. SUMOylation is an important PTM of FOXM1 that regulates its activity, stability, and other PTMs. The functions of FOXM1 SUMOylation are diverse and may regulate its activity in an isoform-specific manner.

Multiple sites of FOXM1 are modified by SUMO-2, and this modification peaks in the M phase, which is consistent with its phosphorylation. This modification blocks the dimerization and relieves the auto-repression of FOXM1, thereby increasing its transcriptional activity [78]. Another SUMOylation of FOXM1 at K463 by SUMO-1 is also required for its transcriptional activity [79].

In contrast, SUMOlyation of FOXM1 at different sites may inhibit its activity. For instance, FOXM1c is modified by SUMO-1 at multiple sites, which promotes the cytoplasmic translocation of FOXM1c and enhances APC/Cdh1-dependent ubiquitin-mediated degradation. This modification subsequently attenuates the transcriptional activity of FOXM1c [80]. This phenomenon has been confirmed with another FOXM1 isoform-FOXM1b [81].

### Other PTMs of FOXM1

FOXM1 is also regulated by acetyltransferases and methyltransferases. For instance, FOXM1 can be acetylated by p300/CBP at lysines K63, K422, K440, K603 and K614, which enhances its transcriptional activity by promoting its DNA binding affinity, protein stability, and phosphorylation sensitivity [82]. Under normoxic conditions, methyltransferase SETD3 specifically binds and methylates FOXM1, which inhibits its activity [83].

In general, we can conclude that the FOXM1 protein can be modified by multiple PTMs, including phosphorylation, ubiquitination, SUMOylation, acetylation, and so on. It has been demonstrated that these PTMs can result in the spatiotemporal control of target protein expression. This may provide new strategies for the modulation of FOXM1 expression utilizing the key enzymes involved in these PTMs.

## Protein/RNA directly interacts with FOXM1 protein

The protein-protein/RNA interactions of FOXM1 are discussed in detail in a recently published review [84]. This article revisits these interactions and focuses on molecules that directly interact with FOXM1 (Table 4).

**Table 4:** proteins/RNA interact with FOXM1

Protein/RNA	Binding site	Effect	Cell cycle	FOXM1 isoform
B55α [61]	^a^	CyclinA CDK2 activity↓	G1	FOXM1c
RB [85]	359/425	Transcriptional activity↓	G1	FOXM1c
p19^ARF^ [86]	688/748	Transcriptional activity↓		FOXM1b
NPM [87]	195/688	Nuclear translocation↑		FOXM1b
CDC25A [89]	C-terminal	CDK1 activity↑		FOXM1
PHGDH [133]	N-terminal	Stabilization↑		FOXM1
MELK [88]	N-terminal	Plk1 activity↑	G2 → M/M	FOXM1
Pin1 [134]	S331,704	Transcriptional activity↑		FOXM1
MTDH [90]	N-terminal	Stabilization↑ transcriptional activity↑		FOXM1b
PVT1(lncRNA) [91]	^a^	Stabilization↑		FOXM1
HSP70 [135]	^a^	Transcriptional activity↓		FOXM1

*CD* central domain

^a^ not clear

↓ Decrease

↑ Increase

→ Transition

In the G1 phase, RB acts as a repressor by binding directly to the central domain of FOXM1, which may recruit NRD to inhibit TAD. Cyclin D1/Cdk4 activates FOXM1 by releasing its TAD from repression by RB, which might lead to deregulated proliferation and cancer [85]. Another tumor suppressor p19^ARF^ interacts directly with the C-terminal (688–748) of FOXM1, and decreases FOXM1 transcriptional activity. At the same time, the interaction also diminishes FOXM1 stimulation of colony formation of U2OS cells, suggesting that p19^ARF^ may be an effective therapeutic inhibitor of FOXM1 transformation function [86].

Several proteins can increase FOXM1 activity by promoting its stabilization, nuclear location, and phosphorylation or inhibiting its ubiquitination. For example, in cancer cells nucleophosmin (NPM) interacts with FOXM1 and their interaction is required for sustaining the level and nucleus localization of FOXM1 [87]. In glioma stem-like cell (GSC), maternal embryonic leucine-zipper kinase (MELK) increases the activity of FOXM1 by interaction with its N-terminus and promoting its phosphorylation by Plk1 [88]. As mentioned above, PLK1 binds and phosphorylates FOXM1 leading to its activation and increased gene expression, which are required for mitotic progression. In addition, cell division cycle 25A (CDC25A) interacts with the C-terminus and enhances CDK1-dependent phosphorylation of FOXM1 [89]. This phosphorylation is required to release the inhibitory function of the NRD during G2 phase (as mentioned above). Another protein, metadherin/astrocyte elevated gene-1 (MTDH), directly interacts with FOXM1 via the N-terminal inhibitory domain of MTDH, and this interaction disrupts the binding of Cdh-1 (mentioned above) to FOXM1, thus protecting FOXM1 from subsequent proteasomal degradation. At the same time, MTDH also binds to FOXM1 target gene promoters and enhances its transcriptional activity. All these interactions promote cell cycle progression, angiogenesis, and cancer cell invasion in vitro and in vivo [90].

As mentioned above, lncRNA can act as an oncogenic factor through the regulation of the transcriptional level of FOXM1. Interestingly, the lncRNA PVT1 can bind to FOXM1 protein and elevate its levels by reducing its degradation and enhancing its stability, subsequently promoting the proliferation and invasion of gastric cancer cells [91]. This implies that noncoding RNA can not only function at the transcriptional level, but also play a role in other processes. The clarification of the interaction binding sites between FOXM1 and other proteins will provide implication for the design of short peptides and small molecular targeting FOXM1.

## Pharmacological inhibition of FOXM1

Significant progress has been accomplished over the last few years in terms of the pharmacological inhibition of FOXM1 in cancer [92–94]. It is apparent, as outlined in the above discussion, that the inhibition of FOXM1 expression (at the levels of transcription, translation, and post-translation) and/or its interactions with target sites (block DBD, nuclear localization, protein-protein interaction) may be an effective way to inhibit FOXM1-mediated biological effects (Table 5). For example, Sp1 directly binds to the promotor of FOXM1 and activates its transcription. Thiazolidinedione (TZD) inhibits FOXM1 expression through downregulation of Sp1, which may negatively regulate tumor cell growth and promote apoptosis [95]. Diarylheptanoids, from medicinal plants, can also suppress FOXM1 and expression of its target genes by suppressing Gli1 in pancreatic cancer cells [96].

**Table 5:** Inhibitors of FOXM1 whose mechanism has been elucidated

Inhibitors	Mechanism	Effect	Cancer cells
Thiazolidinediones	Inhibit Sp1	mRNA exprssion^b^	hepatoma [95]
Diarylheptanoids	Inhibit Gli1	mRNA and protein exprssion^b^	pancreatic cancer [96]
RCM-1	increase ubiquitination	protein degradation^a^	osteosarcoma [99]
Thiostrepton	Interact with FOXM1	binding of FOXM1 to target sites^b^	breast cancer [98]
honokiol	Interact with FOXM1	binding of FOXM1 to target sites^b^	osteosarcoma [100]
FDI-6	Block FOXM1 DBD	binding of FOXM1 to target sites^b^	MCF-7[128]
FOXM1 Apt	target FOXM1 DBD	binding of FOXM1 to target sites^b^	HEK 293 T [136]
Peptide 9R-P201	target FOXM1 DBD	FOXM1 and garget gene expression^b^	HepG2 [137]

^a^: Promoting;

^b^: Inhibition

That said, the biological effects of targeting transcription factors are diversified, and it may not be the best therapeutic solution. The siRNA and ARF peptide target FOXM1 is relatively specific and effective, but the drug-targeted delivery and immune responses may be a major obstacle to be clinical use. Another classical FOXM1 inhibitor thiostrepton, a thiazole antibiotic, inhibits FOXM1 through interacting directly with FOXM1 protein and acting as a proteasome inhibitor [97, 98]. Several recent studies have found that small molecule inhibitors work well to inhibit FOXM1. Sun L et al. identified a small molecule RCM-1 by high-throughput screen, which blocks the nuclear localization and increases the proteasomal degradation of FOXM1 with less effect on other FOX family transcription factors [99]. Another small molecule, honokiol, inhibits FOXM1 by specific binding in a way that is structurally strict [100]. Although much work has been done, there is much more to accomplish to identify specific FOXM1 inhibitors and to validate them in clinical trials. These FOXM1 inhibitors may be used as single agents or in combination with low-dose chemotherapy for cancer treatment.

## FOXM1 in the tumor microenvironment

The tumor microenvironment is created by the tumor and dominated by tumor-induced interactions. Although various immune effector cells are recruited to the tumor site, their anti-tumor functions are suppressed. Infiltrates of inflammatory cells present in human tumors are chronic in nature and are enriched in regulatory T cells (Treg) as well as myeloid suppressor cells (MSC) [101]. Immunotherapeutic strategies, including cancer vaccines, oncolytic viruses, adoptive transfer of ex vivo activated T and natural killer cells, and administration of antibodies or recombinant proteins, are now being described at a breathtaking pace, especially after the clinical application of the monoclonal antibody blocking of cytotoxic T lymphocyte-associated protein 4 (CTLA-4) and programmed cell death protein 1 (PD1) [102]. Few research have revealed the role of FOXM1 in immune cells: FOXM1 may be involved in the determination of induced Tregs (iTreg) versus Teff development during T cell differentiation [103]. Furthermore, latest reports revealed that FOXM1 modulates atherosclerosis by inducing macrophage proliferation [104]. However, little is known about the possible role of FOXM1 in tumor microenvironment. Interestingly, FOXM1 (362–370) (YLVPIQFPV), FOXM1(373–382) (SLVLQPSVKV), and FOXM1(640–649) (GLMDLSTTPL) peptides primed HLA-A2-restricted cytotoxic T lymphocytes (CTLs) in the HLA-A2 transgenic mice, suggesting that FOXM1 may be a suitable target for immunotherapy against cancers. However, the HLAA2-restricted epitopes of FOXM1 identified need to be further clinically tested [105]. As the important role of FOXM1 in cell proliferation and determination of cell fate, more study is needed to reveal the possible role of FOXM1 in the tumor infiltrating immune cells.

## Conclusions and future perspectives

FOXM1 is a crucial regulator of many biological processes and tissues, and dysregulation of FOXM1 can significantly contribute to tumorigenesis and cancer progression. For its potential as a target for cancer therapies, FOXM1 was named the Molecule of the Year in 2010 [106]. Over the past few decades, understanding of the regulation and function of FOXM1 has rapidly increased, providing new insights into the roles of this transcription factor in cancer and other diseases. At the same time, some small molecule inhibitors that target FOXM1 have promising potential as drugs for cancer treatment [107, 108]. However, there are important challenges that limit the translation of promising drugs into clinical practice. Before the entry of FOXM1 inhibitors into clinical trials, more thorough preclinical studies on their anti-tumor efficacy are still needed. In addition, the toxicity of the above drugs should also be fully evaluated. It is not quite clear how the interaction and isoforms switch between FOXM1a and FOXM1b or FOXM1c or FOXM1d. It also remains unclear how the isoforms of FOXM1 interact and what role they may play in the regulation of FOXM1, in disease progression, or in response to relevant therapeutic strategies. Importantly, although the crystal structure of the FOXM1 DNA-recognition domain has been fully identified [7], it is vital that the complete structure of the FOXM1 protein be elucidated. This will be of utmost importance for the discovery of novel FOXM1 inhibitors.

**Fig. 4:**
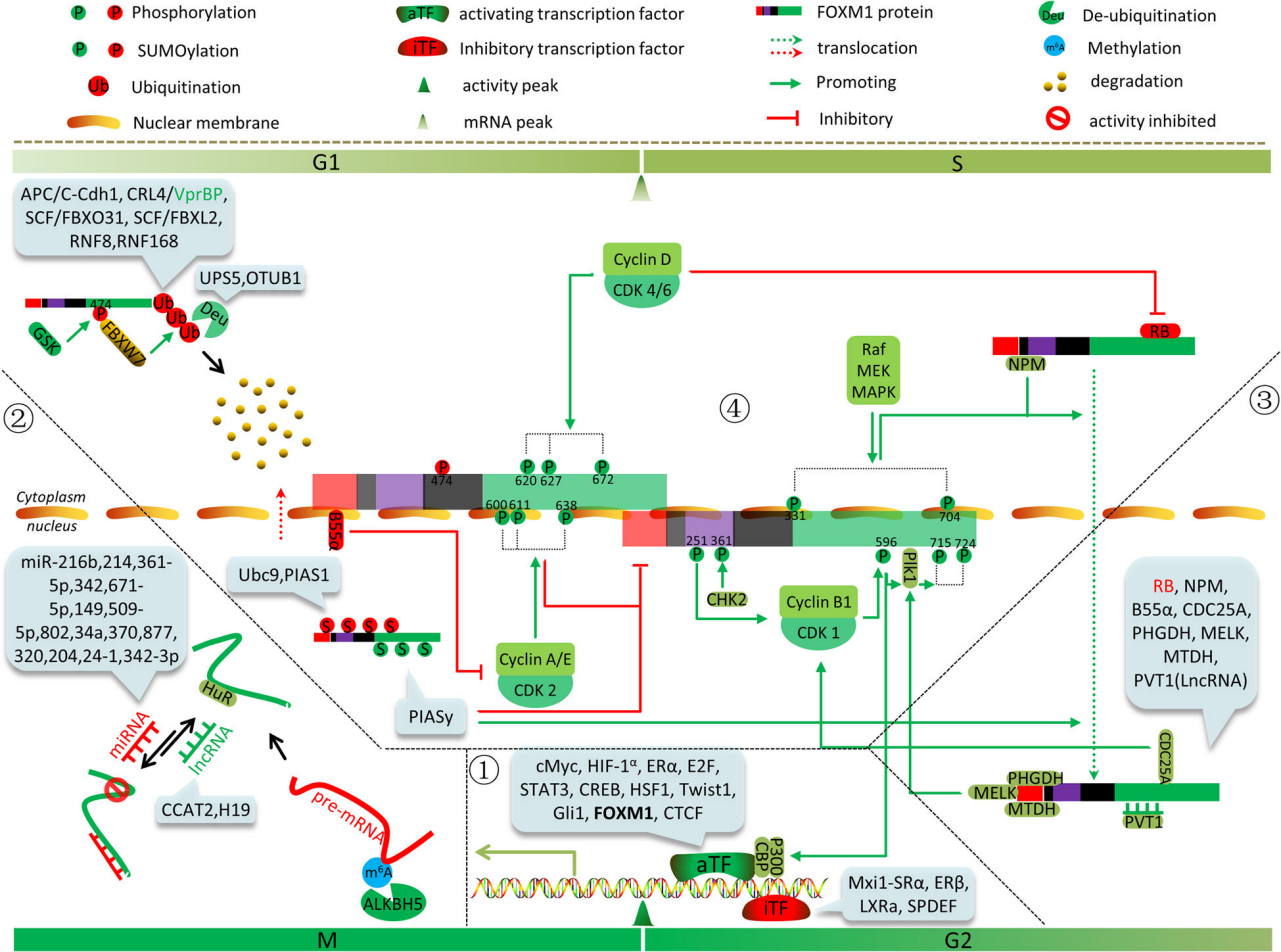
Schematic diagram of FOXM1 regulation network. This schematic diagram attempts to address the regulation of FOXM1 in a spherical way. Green: promotion of FXOM1 expression or transcriptional activity; Red: repression of FOXM1 expression or transcriptional activity. ①.Transcriptional regulation of FOXM1: iTF (inhibitory transcription factor) binds to the promoter region of FOXM1 and inhibits its transcription. aTF (activating transcription factor) binds to the promoter region of FOXM1 and enhances its transcription. ②.Post-transcriptional regulation of FOXM1: m6A methylation of FOXM1 pre-mRNA and the miRNAs binding to 3′UTRs of FOXM1 mRNA and guiding FOXM1 are listed. lncRNAs can act as ceRNAs and block the suppression of FOXM1 by miRNAs. ③. Protein/RNA directly interacts with FOXM1 protein: The interaction of protein/RNA with FOXM1 protein alter the cellular localization, stabilization, or transcriptional activity of FOXM1. ④. Post-translational modifications of FOXM1 protein

In this review, we have summarized many of the activators and repressors that directly interact with or modify FOXM1 at multiple levels and drew the FOXM1-interaction network diagram (Fig. 4). The comprehensive understanding of the regulation of FOXM1 will provide a basis for further investigation, which may provide new potential therapeutic strategies.

## Abbreviations

APC/C: Anaphase-promoting complex-cyclosome
AURKA: Aurora kinase A
CCAT2: Colon cancer-associated transcript 2
CDC25A: Cell division cycle 25A
ceRNAs: Competing endogenous RNAs
Chk2: Checkpoint kinase 2
CRC: Colorectal cancer
CREB: cAMP responsive element-binding protein
CTCF: CCCTC-binding factor
DBD: Forkhead DNA-binding domain
EMT: Epithelial-mesenchymal transition
ERE: Estrogen-response element
FBXW7: F-box/WD repeat-containing protein 7
FDI-6: A small molecule compound
FOXM1 Apt: FOXM1-specific single stranded DNA aptamer
FOXM1: Human Forkhead Box M1
GBC: Gallbladder cancer
Gli1: Glioma-associated oncogene homolog 1
GSC: Glioma stem-like cell
HCC: Hepatocellular carcinoma
HDAC: Histone deacetylases
HIF-1: Hypoxia-inducible factor 1
HSF1: Heat shock factor 1
lncRNAs: Long non-coding RNAs
LXRa: Liver X receptor a
MAPK: Mitogen-activated Protein Kinase
MELK: Maternal embryonic leucine-zipper kinase
miRNAs: MicroRNAs
MnSOD: Manganese-dependent superoxide dismutase
MREs: Response elements
MTDH: Metadherin/astrocyte elevated gene-1
ncRNA: Non-coding RNA
NPM: Nucleophosmin
NRD: N-terminal repressor domain
p19^ARF^: 19-kD alternative reading frame (ARF) protein
Peptide 9R-P201: A peptidomimetics from the phage random library
RCM-1: Robert Costa Memorial drug–1
SETD3: SET domain-containing protein
STAT3: Signal transducer and activator of transcription 3
SUMO: Small Ubiquitin-like Modifier
TAD: C-terminal transactivation domain
TNF-α: Tumor necrosis factor alpha
Twist1: Twist-related protein 1

## References

[1] Clark KL, Halay ED, Lai E, Burley SK (1993). Co-crystal structure of the HNF-3/fork head DNA-recognition motif resembles histone H5. Nature.

[2] Korver W, Roose J, Heinen K, Weghuis DO, de Bruijn D, van Kessel AG, Clevers H (1997). The human TRIDENT/HFH-11/FKHL16 gene: structure, localization, and promoter characterization. Genomics.

[3] Zona S, Bella L, Burton MJ, Nestal de Moraes G, Lam EW-F (2014). FOXM1: an emerging master regulator of DNA damage response and genotoxic agent resistance. Biochim Biophys Acta.

[4] Zhang X, Zhang L, Du Y, Zheng H, Zhang P, Sun Y, Wang Y, Chen J, Ding P, Wang N, Yang C, Huang T, Yao X, Qiao Q, Gu H, Cai G, Cai S, Zhou X, Hu W (2017). A novel FOXM1 isoform, FOXM1D, promotes epithelial-mesenchymal transition and metastasis through ROCKs activation in colorectal cancer. Oncogene.

[5] Park HJ, Wang Z, Costa RH, Tyner A, Lau LF, Raychaudhuri P (2008). An N-terminal inhibitory domain modulates activity of FoxM1 during cell cycle. Oncogene.

[6] Anders L, Ke N, Hydbring P, Choi YJ, Widlund HR, Chick JM, Zhai H, Vidal M, Gygi SP, Braun P, Sicinski P (2011). A systematic screen for CDK4/6 substrates links FOXM1 phosphorylation to senescence suppression in cancer cells. Cancer Cell.

[7] Littler DR, Alvarezfernández M, Stein A, Hibbert RG, Heidebrecht T, Aloy P, Medema RH, Perrakis A (2010). Structure of the FoxM1 DNA-recognition domain bound to a promoter sequence. Nucleic Acids Res.

[8] Besharat ZM, Abballe L, Cicconardi F, Bhutkar A, Grassi L, Le Pera L, Moretti M, Chinappi M, D’Andrea D, Mastronuzzi A. Foxm1 controls a pro-stemness microRNA network in neural stem cells. Sci Rep. 2018;8 10.1038/s41598-018-21876-y. 10.1038/s41598-018-21876-y PMC5824884 29476172

[9] Wang D, Hu G, Du Y, Zhang C, Lu Q, Lv N, Luo S (2017). Aberrant activation of hedgehog signaling promotes cell proliferation via the transcriptional activation of forkhead box M1 in colorectal cancer cells. J Exp Clin Cancer Res.

[10] Teh M-T, Wong S-T, Neill GW, Ghali LR, Philpott MP, Quinn AG (2002). FOXM1 is a downstream target of Gli1 in basal cell carcinomas. Cancer Res.

[11] Nandi D, Cheema PS, Jaiswal N, Nag A. FoxM1: repurposing an oncogene as a biomarker. Semin Cancer Biol. 2017; 10.1016/j.semcancer.2017.08.009. 10.1016/j.semcancer.2017.08.009 28855104

[12] Wierstra I (2013). FOXM1 (Forkhead box M1) in tumorigenesis: overexpression in human cancer, implication in tumorigenesis, oncogenic functions, tumor-suppressive properties, and target of anticancer therapy. Adv Cancer Res.

[13] Tassi RA, Todeschini P, Siegel ER, Calza S, Cappella P, Ardighieri L, Cadei M, Bugatti M, Romani C, Bandiera E, Zanotti L, Tassone L, Guarino D, Santonocito C, Capoluongo ED, Beltrame L, Erba E, Marchini S, D’Incalci M, Donzelli C, Santin AD, Pecorelli S, Sartori E, Bignotti E, Odicino F, Ravaggi A (2017). FOXM1 expression is significantly associated with chemotherapy resistance and adverse prognosis in non-serous epithelial ovarian cancer patients. J Exp Clin Cancer Res.

[14] Abdeljaoued S, Bettaieb I, Nasri M, Adouni O, Goucha A, El Amine O, Boussen H, Rahal K, Gamoudi A (2017). Overexpression of FOXM1 is a potential prognostic marker in male breast Cancer. Oncol Res Treat.

[15] Liu Y, Liu Y, Yuan B, Yin L, Peng Y, Yu X, Zhou W, Gong Z, Liu J, He L, Li X (2017). FOXM1 promotes the progression of prostate cancer by regulating PSA gene transcription. Oncotarget.

[16] Egawa M, Yoshida Y, Ogura S, Kurahashi T, Kizu T, Furuta K, Kamada Y, Chatani N, Hamano M, Kiso S, Hikita H, Tatsumi T, Eguchi H, Nagano H, Doki Y, Mori M, Takehara T (2017). Increased expression of Forkhead box M1 transcription factor is associated with clinicopathological features and confers a poor prognosis in human hepatocellular carcinoma. Hepatol Res.

[17] Ito T, Kohashi K, Yamada Y, Iwasaki T, Maekawa A, Kuda M, Hoshina D, Abe R, Furue M, Oda Y (2016). Prognostic significance of Forkhead box M1 (FOXM1) expression and antitumor effect of FOXM1 inhibition in Angiosarcoma. J Cancer.

[18] Zhang H, Zhong H, Li L, Ji W, Zhang X (2016). Overexpressed transcription factor FOXM1 contributes to the progression of colorectal cancer. Mol Med Rep.

[19] Ito T, Kohashi K, Yamada Y, Maekawa A, Kuda M, Furue M, Oda Y (2016). Prognostic significance of forkhead box M1 (FoxM1) expression and antitumour effect of FoxM1 inhibition in melanoma. Histopathology.

[20] Kong F-F, Qu Z-Q, Yuan H-H, Wang J-Y, Zhao M, Guo Y-H, Shi J, Gong X-D, Zhu Y-L, Liu F, Zhang W-Y, Jiang B (2014). Overexpression of FOXM1 is associated with EMT and is a predictor of poor prognosis in non-small cell lung cancer. Oncol Rep.

[21] Okada K, Fujiwara Y, Takahashi T, Nakamura Y, Takiguchi S, Nakajima K, Miyata H, Yamasaki M, Kurokawa Y, Mori M, Doki Y (2013). Overexpression of forkhead box M1 transcription factor (FOXM1) is a potential prognostic marker and enhances chemoresistance for docetaxel in gastric cancer. Ann Surg Oncol.

[22] Zhong S, Zhou A, Qi F, Li Z, Yu Z, Lu Y, Liu X (2017). Downregulating forkhead box M1 inhibits proliferation by inhibiting autophagy in the sw480 cell line. Biomed Rep.

[23] Zhang J, Niu Y, Huang C (2017). Role of FoxM1 in the progression and epithelial to mesenchymal transition of gastrointestinal Cancer. Recent Pat Anticancer Drug Discov.

[24] Wang K, Zhu X, Zhang K, Zhu L, Zhou F (2016). FoxM1 inhibition enhances chemosensitivity of docetaxel-resistant A549 cells to docetaxel via activation of JNK/mitochondrial pathway. Acta Biochim Biophys Sin Shanghai.

[25] Yu C, Chen L, Yie L, Wei L, Wen T, Liu Y, Chen H (2015). Targeting FoxM1 inhibits proliferation, invasion and migration of nasopharyngeal carcinoma through the epithelialto-mesenchymal transition pathway. Oncol Rep.

[26] Jiang L, Wang P, Chen L, Chen H (2014). Down-regulation of FoxM1 by thiostrepton or small interfering RNA inhibits proliferation, transformation ability and angiogenesis, and induces apoptosis of nasopharyngeal carcinoma cells. Int J Clin Exp Pathol.

[27] Li L, Wu D, Yu Q, Li L, Wu P (2017). Prognostic value of FOXM1 in solid tumors: a systematic review and meta-analysis. Oncotarget.

[28] Chandrashekar DS, Bashel B, Sah B, Creighton CJ, Poncerodriguez I, Bvsk C, Varambally S (2017). UALCAN: a portal for facilitating tumor subgroup gene expression and survival analyses. Neoplasia.

[29] Koo C-Y, Muir KW, Lam EW-F (2012). FOXM1: from cancer initiation to progression and treatment. Biochim Biophys Acta.

[30] Zhang B, Zhang Y, Zou X, Chan AWH, Zhang R, Lee TK-W, Liu H, Lau EY-T, Ho NP-Y, Lai PBS, Cheung Y-S, K.-F. To, Wong HK, Choy KW, Keng VW, Chow LMC, Chan KKY, Cheng AS, Ko BCB. CTCF-FOXM1 axis regulates tumor growth and metastasis in hepatocellular carcinoma. J Pathol. 2017; 10.1002/path.4976. 10.1002/path.4976 PMC5725705 28862757

[31] Chen P-M, Wu T-C, Shieh S-H, Wu Y-H, Li M-C, Sheu G-T, Cheng Y-W, Chen C-Y, Lee H (2013). MnSOD promotes tumor invasion via upregulation of FoxM1-MMP2 axis and related with poor survival and relapse in lung adenocarcinomas. Mol Cancer Res.

[32] Mencalha AL, Binato R, Ferreira GM, Du Rocher B, Abdelhay E (2012). Forkhead box M1 (FoxM1) gene is a new STAT3 transcriptional factor target and is essential for proliferation, survival and DNA repair of K562 cell line. PLoS One.

[33] Xia L, Huang W, Tian D, Zhu H, Zhang Y, Hu H, Fan D, Nie Y, Wu K (2012). Upregulated FoxM1 expression induced by hepatitis B virus X protein promotes tumor metastasis and indicates poor prognosis in hepatitis B virus-related hepatocellular carcinoma. J Hepatol.

[34] Millour J, de Olano N, Horimoto Y, Monteiro LJ, Langer JK, Aligue R, Hajji N, Lam EWF (2011). ATM and p53 regulate FOXM1 expression via E2F in breast cancer epirubicin treatment and resistance. Mol Cancer Ther.

[35] Qian J, Luo Y, Gu X, Zhan W, Wang X (2013). Twist1 promotes gastric cancer cell proliferation through up-regulation of FoxM1. PLoS One.

[36] Xia L-M, Huang W-J, Wang B, Liu M, Zhang Q, Yan W, Zhu Q, Luo M, Zhou Z-Z, Tian D-A (2009). Transcriptional up-regulation of FoxM1 in response to hypoxia is mediated by HIF-1. J Cell Biochem.

[37] Xia L, Mo P, Huang W, Zhang L, Wang Y, Zhu H, Tian D, Liu J, Chen Z, Zhang Y, Chen Z, Hu H, Fan D, Nie Y, Wu K (2012). The TNF-alpha/ROS/HIF-1-induced upregulation of FoxMI expression promotes HCC proliferation and resistance to apoptosis. Carcinogenesis.

[38] Dai B, Gong A, Jing Z, Aldape KD, Kang S-H, Sawaya R, Huang S (2013). Forkhead box M1 is regulated by heat shock factor 1 and promotes glioma cells survival under heat shock stress. J Biol Chem.

[39] Hu C, Liu D, Zhang Y, Lou G, Huang G, Chen B, Shen X, Gao M, Gong W, Zhou P, Dai S, Zeng Y, He F (2014). LXRalpha-mediated downregulation of FOXM1 suppresses the proliferation of hepatocellular carcinoma cells. Oncogene.

[40] Barsotti AM, Prives C (2009). Pro-proliferative FoxM1 is a target of p53-mediated repression. Oncogene.

[41] Kurinna S, Stratton SA, Coban Z, Schumacher JM, Grompe M, Duncan AW, Barton MC (2013). p53 regulates a mitotic transcription program and determines ploidy in normal mouse liver. Hepatology.

[42] Millour J, Constantinidou D, Stavropoulou AV, Wilson MSC, Myatt SS, Kwok JM-M, Sivanandan K, Coombes RC, Medema RH, Hartman J, Lykkesfeldt AE, Lam EW-F (2010). FOXM1 is a transcriptional target of ERalpha and has a critical role in breast cancer endocrine sensitivity and resistance. Oncogene.

[43] Horimoto Y, Hartman J, Millour J, Pollock S, Olmos Y, Ho K-K, Coombes RC, Poutanen M, Makela SI, El-Bahrawy M, Speirs V, Lam EW-F (2011). ERbeta1 represses FOXM1 expression through targeting ERalpha to control cell proliferation in breast cancer. Am J Pathol.

[44] Blanco-Bose WE, Murphy MJ, Ehninger A, Offner S, Dubey C, Huang W, Moore DD, Trumpp A (2008). C-Myc and its target FoxM1 are critical downstream effectors of constitutive androstane receptor (CAR) mediated direct liver hyperplasia. Hepatology.

[45] Delpuech O, Griffiths B, East P, Essafi A, Lam EW-F, Burgering B, Downward J, Schulze A (2007). Induction of Mxi1-SR alpha by FOXO3a contributes to repression of Myc-dependent gene expression. Mol Cell Biol.

[46] Halasi M, Gartel AL (2009). A novel mode of FoxM1 regulation: positive auto-regulatory loop. Cell Cycle.

[47] Cheng X-H, Black M, Ustiyan V, Le T, Fulford L, Sridharan A, Medvedovic M, Kalinichenko VV, Whitsett JA, Kalin TV (2014). SPDEF inhibits prostate carcinogenesis by disrupting a positive feedback loop in regulation of the Foxm1 oncogene. PLoS Genet.

[48] Gartel AL (2015). Targeting FOXM1 auto-regulation in cancer. Cancer Biol Ther.

[49] Yang N, Wang C, Wang Z, Zona S, Lin S-X, Wang X, Yan M, Zheng F-M, Li S-S, Xu B, Bella L, Yong J-S, Lam EW-F, Liu Q (2017). FOXM1 recruits nuclear aurora kinase a to participate in a positive feedback loop essential for the self-renewal of breast cancer stem cells. Oncogene.

[50] Xie X, Lu J, Kulbokas EJ, Golub TR, Mootha V, Lindblad-Toh K, Lander ES, Kellis M (2005). Systematic discovery of regulatory motifs in human promoters and 3’ UTRs by comparison of several mammals. Nature.

[51] Xu Z, Yan Y, Qian L, Gong Z (2017). Long non-coding RNAs act as regulators of cell autophagy in diseases (Review). Oncol Rep.

[52] Batista PJ, Chang HY (2013). Long noncoding RNAs: cellular address codes in development and disease. Cell.

[53] Yamamura S, Imai-Sumida M, Tanaka Y, Dahiya R. Interaction and cross-talk between non-coding RNAs. Cell Mol Life Sci. 2017; 10.1007/s00018-017-2626-6. 10.1007/s00018-017-2626-6 PMC5765200 28840253

[54] Wang J-M, Ju B-H, Pan C-J, Gu Y, Li M-Q, Sun L, Xu Y-Y, Yin L-R (2017). MiR-214 inhibits cell migration, invasion and promotes the drug sensitivity in human cervical cancer by targeting FOXM1. Am J Transl Res.

[55] Liu X, Xie T, Mao X, Xue L, Chu X, Chen L (2016). MicroRNA-149 increases the sensitivity of colorectal Cancer cells to 5-fluorouracil by targeting Forkhead box transcription factor FOXM1. Cell Physiol Biochem.

[56] Li X-R, Chu H-J, Lv T, Wang L, Kong S-F, Dai S-Z (2014). miR-342-3p suppresses proliferation, migration and invasion by targeting FOXM1 in human cervical cancer. FEBS Lett.

[57] Wang S-H, Ma F, Tang Z-H, Wu X-C, Cai Q, Zhang M-D, Weng M-Z, Zhou D, Wang J-D, Quan Z-W (2016). Long non-coding RNA H19 regulates FOXM1 expression by competitively binding endogenous miR-342-3p in gallbladder cancer. J Exp Clin Cancer Res.

[58] Chen F, Bai G, Li Y, Feng Y, Wang L (2017). A positive feedback loop of long noncoding RNA CCAT2 and FOXM1 promotes hepatocellular carcinoma growth. Am J Cancer Res.

[59] Chen Y-J, Dominguez-Brauer C, Wang Z, Asara JM, Costa RH, Tyner AL, Lau LF, Raychaudhuri P (2009). A conserved phosphorylation site within the forkhead domain of FoxM1B is required for its activation by cyclin-CDK1. J Biol Chem.

[60] Ma RYM, Tong THK, Cheung AMS, Tsang ACC, Leung WY, Yao K-M (2005). Raf/MEK/MAPK signaling stimulates the nuclear translocation and transactivating activity of FOXM1c. J Cell Sci.

[61] Alvarez-Fernandez M, Halim VA, Aprelia M, Laoukili J, Mohammed S, Medema RH (2011). Protein phosphatase 2A (B55alpha) prevents premature activation of forkhead transcription factor FoxM1 by antagonizing cyclin a/cyclin-dependent kinase-mediated phosphorylation. J Biol Chem.

[62] Luscher-Firzlaff JM, Lilischkis R, Luscher B (2006). Regulation of the transcription factor FOXM1c by cyclin E/CDK2. FEBS Lett.

[63] Laoukili J, Alvarez M, Meijer LAT, Stahl M, Mohammed S, Kleij L, Heck AJR, Medema RH (2008). Activation of FoxM1 during G2 requires cyclin a/Cdk-dependent relief of autorepression by the FoxM1 N-terminal domain. Mol Cell Biol.

[64] Fu Z, Malureanu L, Huang J, Wang W, Li H, van Deursen JM, Tindall DJ, Chen J (2008). Plk1-dependent phosphorylation of FoxM1 regulates a transcriptional programme required for mitotic progression. Nat Cell Biol.

[65] Major ML, Lepe R, Costa RH (2004). Forkhead box M1B transcriptional activity requires binding of Cdk-cyclin complexes for phosphorylation-dependent recruitment of p300/CBP coactivators. Mol Cell Biol.

[66] Tan Y, Raychaudhuri P, Costa RH (2007). Chk2 mediates stabilization of the FoxM1 transcription factor to stimulate expression of DNA repair genes. Mol Cell Biol.

[67] Chen Y, Li Y, Xue J, Gong A, Yu G, Zhou A, Lin K, Zhang S, Zhang N, Gottardi CJ, Huang S (2016). Wnt-induced deubiquitination FoxM1 ensures nucleus beta-catenin transactivation. EMBO J.

[68] Zhang J, Yuan C, Wu J, Elsayed Z, Fu Z (2015). Polo-like kinase 1-mediated phosphorylation of Forkhead box protein M1b antagonizes its SUMOylation and facilitates its mitotic function. J Biol Chem.

[69] Newton K, Matsumoto ML, Wertz IE, Kirkpatrick DS, Lill JR, Tan J, Dugger D, Gordon N, Sidhu SS, Fellouse FA, Komuves L, French DM, Ferrando RE, Lam C, Compaan D, Yu C, Bosanac I, Hymowitz SG, Kelley RF, Dixit VM (2008). Ubiquitin chain editing revealed by polyubiquitin linkage-specific antibodies. Cell.

[70] Pfleger CM, Kirschner MW (2000). The KEN box: an APC recognition signal distinct from the D box targeted by Cdh1. Genes Dev.

[71] Park HJ, Costa RH, Lau LF, Tyner AL, Raychaudhuri P (2008). Anaphase-promoting complex/cyclosome-CDH1-mediated proteolysis of the forkhead box M1 transcription factor is critical for regulated entry into S phase. Mol Cell Biol.

[72] Laoukili J, Alvarez-Fernandez M, Stahl M, Medema RH (2008). FoxM1 is degraded at mitotic exit in a Cdh1-dependent manner. Cell Cycle.

[73] Wang X, Arceci A, Bird K, Mills CA, Choudhury R, Kernan JL, Zhou C, Bae-Jump V, Bowers A, Emanuele MJ. VprBP/DCAF1 regulates the degradation and nonproteolytic activation of the cell cycle transcription factor FoxM1. Mol Cell Biol. 2017;37 10.1128/MCB.00609-16. 10.1128/MCB.00609-16 PMC5472828 28416635

[74] Jeffery JM, Kalimutho M, Johansson P, Cardenas DG, Kumar R, Khanna KK (2017). FBXO31 protects against genomic instability by capping FOXM1 levels at the G2/M transition. Oncogene.

[75] Kongsema M, Zona S, Karunarathna U, Cabrera E, Man EPS, Yao S, Shibakawa A, Khoo U-S, Medema RH, Freire R, Lam EW-F (2016). RNF168 cooperates with RNF8 to mediate FOXM1 ubiquitination and degradation in breast cancer epirubicin treatment. Oncogene.

[76] Karunarathna U, Kongsema M, Zona S, Gong C, Cabrera E, Gomes AR, Man EPS, Khongkow P, Tsang JW-H, Khoo U-S, Medema RH, Freire R, Lam EW-F (2016). OTUB1 inhibits the ubiquitination and degradation of FOXM1 in breast cancer and epirubicin resistance. Oncogene.

[77] Wang Y, Zhou X, Xu M, Weng W, Zhang Q, Yang Y, Wei P, Du X (2016). OTUB1-catalyzed deubiquitination of FOXM1 facilitates tumor progression and predicts a poor prognosis in ovarian cancer. Oncotarget.

[78] Schimmel J, Eifler K, Sigurethsson JO, Cuijpers SAG, Hendriks IA, Verlaan-de Vries M, Kelstrup CD, Francavilla C, Medema RH, Olsen JV, Vertegaal ACO (2014). Uncovering SUMOylation dynamics during cell-cycle progression reveals FoxM1 as a key mitotic SUMO target protein. Mol Cell.

[79] Wang C-M, Liu R, Wang L, Nascimento L, Brennan VC, Yang W-H (2014). SUMOylation of FOXM1B alters its transcriptional activity on regulation of MiR-200 family and JNK1 in MCF7 human breast cancer cells. Int J Mol Sci.

[80] Myatt SS, Kongsema M, Man CW-Y, Kelly DJ, Gomes AR, Khongkow P, Karunarathna U, Zona S, Langer JK, Dunsby CW, Coombes RC, French PM, Brosens JJ, Lam EW-F (2014). SUMOylation inhibits FOXM1 activity and delays mitotic transition. Oncogene.

[81] Jaiswal N, John R, Chand V, Nag A (2015). Oncogenic human papillomavirus 16E7 modulates SUMOylation of FoxM1b. Int J Biochem Cell Biol.

[82] Lv C, Zhao G, Sun X, Wang P, Xie N, Luo J, Tong T. Acetylation of FOXM1 is essential for its transactivation and tumor growth stimulation. Oncotarget. 2016;7 10.18632/oncotarget.11332. 10.18632/oncotarget.11332 PMC5312389 27542221

[83] Cohn O, Feldman M, Weil L, Kublanovsky M, Levy D. Chromatin associated SETD3 negatively regulates VEGF expression. Nat Publ Gr. 2016:1–10. 10.1038/srep37115. 10.1038/srep37115 PMC5109252 27845446

[84] Gartel AL (2017). FOXM1 in Cancer: interactions and vulnerabilities. Cancer Res.

[85] Wierstra I, Alves J (2006). Transcription factor FOXM1c is repressed by RB and activated by cyclin D1/Cdk4. Biol Chem.

[86] Kalinichenko VV, Major ML, Wang X, Petrovic V, Kuechle J, Yoder HM, Dennewitz MB, Shin B, Datta A, Raychaudhuri P, Costa RH (2004). Foxm1b transcription factor is essential for development of hepatocellular carcinomas and is negatively regulated by the p19 ARF tumor suppressor. Genes Dev.

[87] Bhat UG, Jagadeeswaran R, Halasi M, Gartel AL (2011). Nucleophosmin interacts with FOXM1 and modulates the level and localization of FOXM1 in human cancer cells. J Biol Chem.

[88] Joshi K, Banasavadi-Siddegowda Y, Mo X, Kim S-H, Mao P, Kig C, Nardini D, Sobol RW, Chow LML, Kornblum HI, Waclaw R, Beullens M, Nakano I (2013). MELK-dependent FOXM1 phosphorylation is essential for proliferation of glioma stem cells. Stem Cells.

[89] Sullivan C, Liu Y, Shen J, Curtis A, Newman C, Hock JM, Li X (2012). Novel interactions between FOXM1 and CDC25A regulate the cell cycle. PLoS One.

[90] Yang L, He K, Yan S, Yang Y, Gao X, Zhang M, Xia Z, Huang Z, Huang S, Zhang N (2017). Metadherin/astrocyte elevated gene-1 positively regulates the stability and function of forkhead box M1 during tumorigenesis. Neuro-Oncology.

[91] Xu M-D, Wang Y, Weng W, Wei P, Qi P, Zhang Q, Tan C, Ni S-J, Dong L, Yang Y, Lin W, Xu Q, Huang D, Huang Z, Ma Y, Zhang W, Sheng W, Du X (2017). A positive feedback loop of lncRNA-PVT1 and FOXM1 facilitates gastric Cancer growth and invasion. Clin Cancer Res.

[92] Mónica AF, Medema RH (2013). Novel functions of FoxM1: from molecular mechanisms to cancer therapy. Front Oncol.

[93] Halasi M, Gartel AL (2013). Targeting FOXM1 in cancer. Biochem Pharmacol.

[94] Wierstra I. FOXM1 (Forkhead box M1) in tumorigenesis. Overexpression in human cancer, implication in tumorigenesis, oncogenic functions, tumor-suppressive properties, and target of anticancer therapy. 1st ed: Elsevier Inc; 2013. 10.1016/B978-0-12-407190-2.00016-2. 10.1016/B978-0-12-407190-2.00016-2 23870513

[95] Petrovic V, Costa RH, Lau LF, Raychaudhuri P, Tyner AL (2010). Negative regulation of the oncogenic transcription factor FoxM1 by thiazolidinediones and mithramycin. Cancer Biol Ther.

[96] Dong GZ, Ji HJ, Lee YI, Lee SY, Zhao HY, Jeon R, Lee HJ, Ryu JH (2017). Diarylheptanoids suppress proliferation of pancreatic cancer PANC-1 cells through modulating shh-Gli-FoxM1 pathway. Arch Pharm Res.

[97] Gartel AL (2013). Thiazole antibiotics Siomycin a and Thiostrepton inhibit the transcriptional activity of FOXM1. Front Oncol.

[98] Hegde NS, Sanders DA, Rodriguez R, Balasubramanian S (2011). The transcription factor FOXM1 is a cellular target of the natural product thiostrepton. Nat Chem.

[99] Sun L, Ren X, Wang IC, Pradhan A, Zhang Y, Flood HM, Han B, Whitsett JA, Kalin TV, Kalinichenko VV (2017). The FOXM1 inhibitor RCM-1 suppresses goblet cell metaplasia and prevents IL-13 and STAT6 signaling in allergen-exposed mice. Sci Signal.

[100] Halasi M, Hitchinson B, Shah BN, Váraljai R, Khan I, Benevolenskaya EV, Gaponenko V, Arbiser JL, Gartel AL (2018). Honokiol is a FOXM1 antagonist. Cell Death Dis.

[101] Whiteside TL (2008). The tumor microenvironment and its role in promoting tumor growth. Oncogene.

[102] Farkona S, Diamandis EP, Blasutig IM (2016). Cancer immunotherapy: the beginning of the end of cancer?. BMC Med.

[103] Prots I, Skapenko A, Lipsky PE, Schulzekoops H (2011). Analysis of the transcriptional Program of developing induced regulatory T cells. PLoS One.

[104] M.C. Gage, N. Bécares, R. Louie, K.E. Waddington, Y. Zhang, T.H. Tittanegro, S. Rodr’\iguez-Lorenzo, A. Jathanna, B. Pourcet, O.M. Pello, J. V la Rosa, A. Castrillo, I. Pineda-Torra, Disrupting LXRα phosphorylation promotes FoxM1 expression and modulates atherosclerosis by inducing macrophage proliferation, Proc Natl Acad Sci (2018). doi:10.1073/pnas.1721245115. 10.1073/pnas.1721245115 PMC6048484 29950315

[105] Yokomine K, Senju S, Nakatsura T, Irie A, Hayashida Y, Ikuta Y, Harao M, Imai K, Baba H, Iwase H (2010). The forkhead box M1 transcription factor as a candidate of target for anti-cancer immunotherapy, Int. J Cancer J Int Du Cancer.

[106] Teh MT. Wins Molecule of the Year 2010. 2010. http://ismcbbpr.synthasite.com/molyearnews/foxm1-wins-molecule-of-the-year-2010.

[107] Gormally MV, Dexheimer TS, Marsico G, Sanders DA, Lowe C, Matak-Vinkovic D, Michael S, Jadhav A, Rai G, Maloney DJ, Simeonov A, Balasubramanian S (2014). Suppression of the FOXM1 transcriptional programme via novel small molecule inhibition. Nat Commun.

[108] Gartel AL (2008). FoxM1 inhibitors as potential anticancer drugs. Expert Opin Ther Targets.

[109] Fernandez PC, Frank SR, Wang L, Schroeder M, Liu S, Greene J, Cocito A, Amati B (2003). Genomic targets of the human c-Myc protein. Genes Dev.

[110] Pan H, Zhu Y, Wei W, Shao S, Rui X (2018). Transcription factor FoxM1 is the downstream target of c-Myc and contributes to the development of prostate cancer. World J Surg Oncol.

[111] McGovern UB, Francis RE, Peck B, Guest SK, Wang J, Myatt SS, Krol J, Kwok JM-M, Polychronis A, Coombes RC, Lam EW-F (2009). Gefitinib (Iressa) represses FOXM1 expression via FOXO3a in breast cancer. Mol Cancer Ther.

[112] Pandit B, Halasi M, Gartel AL (2009). p53 negatively regulates expression of FoxM1. Cell Cycle.

[113] Zhang T, Ma G, Zhang Y, Huo H, Zhao Y (2017). miR-216b inhibits glioma cell migration and invasion through suppression of FoxM1. Oncol Rep.

[114] Sun M, Wang X, Tu C, Wang S, Qu J, Xiao S. microRNA-216b inhibits cell proliferation and migration in human melanoma by targeting FOXM1 in vitro and in vivo. Cell Biol Int. 2017; 10.1002/cbin.10754. 10.1002/cbin.10754 28225180

[115] Zheng W-W, Zhou J, Zhang C-H, Liu X-S (2016). MicroRNA-216b is downregulated in hepatocellular carcinoma and inhibits HepG2 cell growth by targeting Forkhead box protein M1. Eur Rev Med Pharmacol Sci.

[116] Hou XW, Sun X, Yu Y, Zhao HM, Yang ZJ, Wang X, Cao XC (2017). miR-361-5p suppresses lung cancer cell lines progression by targeting FOXM1. Neoplasma.

[117] Weng W, Okugawa Y, Toden S, Toiyama Y, Kusunoki M, Goel A (2016). FOXM1 and FOXQ1 are promising prognostic biomarkers and novel targets of tumor-suppressive miR-342 in human colorectal Cancer. Clin Cancer Res.

[118] Tan X, Fu Y, Chen L, Lee W, Lai Y, Rezaei K, Tabbara S, Latham P, Teal CB, Man Y-G, Siegel RS, Brem RF, Fu SW (2016). miR-671-5p inhibits epithelial-to-mesenchymal transition by downregulating FOXM1 expression in breast cancer. Oncotarget.

[119] Y. Ke, W. Zhao, J. Xiong, R. Cao, miR-149 inhibits non-small-cell lung Cancer cells EMT by targeting FOXM1., Biochem Res Int 2013 (2013) 506731. doi:10.1155/2013/506731. 10.1155/2013/506731 PMC3671264 23762558

[120] Ma N, Zhang W, Qiao C, Luo H, Zhang X, Liu D, Zang S, Zhang L, Bai J (2016). The tumor suppressive role of MiRNA-509-5p by targeting FOXM1 in non-small cell lung Cancer. Cell Physiol Biochem.

[121] Yuan F, Wang W (2015). MicroRNA-802 suppresses breast cancer proliferation through downregulation of FoxM1. Mol Med Rep.

[122] Xu X, Chen W, Miao R, Zhou Y, Wang Z, Zhang L, Wan Y, Dong Y, Qu K, Liu C (2015). miR-34a induces cellular senescence via modulation of telomerase activity in human hepatocellular carcinoma by targeting FoxM1/c-Myc pathway. Oncotarget.

[123] Duan N, Hu X, Yang X, Cheng H, Zhang W (2015). MicroRNA-370 directly targets FOXM1 to inhibit cell growth and metastasis in osteosarcoma cells. Int J Clin Exp Pathol.

[124] Yungang W, Xiaoyu L, Pang T, Wenming L, Pan X (2014). miR-370 targeted FoxM1 functions as a tumor suppressor in laryngeal squamous cell carcinoma (LSCC). Biomed Pharmacother.

[125] Feng Y, Wang L, Zeng J, Shen L, Liang X, Yu H, Liu S, Liu Z, Sun Y, Li W, Chen C, Jia J (2013). FoxM1 is overexpressed in helicobacter pylori-induced gastric carcinogenesis and is negatively regulated by miR-370. Mol Cancer Res.

[126] Huang X, Qin J, Lu S (2015). Up-regulation of miR-877 induced by paclitaxel inhibits hepatocellular carcinoma cell proliferation though targeting FOXM1. Int J Clin Exp Pathol.

[127] Wan L-Y, Deng J, Xiang X-J, Zhang L, Yu F, Chen J, Sun Z, Feng M, Xiong J-P (2015). miR-320 enhances the sensitivity of human colon cancer cells to chemoradiotherapy in vitro by targeting FOXM1. Biochem Biophys Res Commun.

[128] Li T, Ma J, Han X, Jia Y, Yuan H, Shui S, Guo D (2018). MicroRNA-320 enhances Radiosensitivity of glioma through down-regulation of Sirtuin type 1 by directly targeting Forkhead box protein M1. Transl Oncol.

[129] Sun Y, Yu X, Bai Q (2015). miR-204 inhibits invasion and epithelial-mesenchymal transition by targeting FOXM1 in esophageal cancer. Int J Clin Exp Pathol.

[130] Inoguchi S, Seki N, Chiyomaru T, Ishihara T, Matsushita R, Mataki H, Itesako T, Tatarano S, Yoshino H, Goto Y, Nishikawa R, Nakagawa M, Enokida H (2014). Tumour-suppressive microRNA-24-1 inhibits cancer cell proliferation through targeting FOXM1 in bladder cancer. FEBS Lett.

[131] Park TJ, Kim JY, Oh SP, Kang SY, Kim BW, Wang HJ, Song KY, Kim HC, Lim IK (2008). TIS21 negatively regulates hepatocarcinogenesis by disruption of cyclin B1-Forkhead box M1 regulation loop. Hepatology.

[132] Li L, Pan D, Chen H, Zhang L, Xie W (2016). F-box protein FBXL2 inhibits gastric cancer proliferation by ubiquitin-mediated degradation of forkhead box M1. FEBS Lett.

[133] Liu J, Guo S, Li Q, Yang L, Xia Z, Zhang L, Huang Z, Zhang N (2013). Phosphoglycerate dehydrogenase induces glioma cells proliferation and invasion by stabilizing forkhead box M1. J Neuro-Oncol.

[134] Kruiswijk F, Hasenfuss SC, Sivapatham R, Baar MP, Putavet D, Naipal KAT, van den Broek NJF, Kruit W, van der Spek PJ, van Gent DC, Brenkman AB, Campisi J, Burgering BMT, Hoeijmakers JHJ, de Keizer PLJ (2016). Targeted inhibition of metastatic melanoma through interference with Pin1-FOXM1 signaling. Oncogene.

[135] Halasi M, Varaljai R, Benevolenskaya E, Gartel AL (2016). A novel function of molecular chaperone HSP70: SUPPRESSION OF ONCOGENIC FOXM1 AFTER PROTEOTOXIC STRESS. J Biol Chem.

[136] Xiang Q, Tan G, Jiang X, Wu K, Tan W, Tan Y (2017). Suppression of FOXM1 transcriptional activities via a single-stranded DNA aptamer generated by SELEX. Sci Rep.

[137] Bi Z, Liu W, Ding R, Wu Y, Dou R, Zhang W, Xue Y, Liu X, Xiong L, Guo Z (2016). A novel peptide, 9R-P201, strongly inhibits the viability, proliferation and migration of liver cancer HepG2 cells and induces apoptosis by down-regulation of FoxM1 expression. Eur J Pharmacol.

